# Editorial: Intravital Microscopy Imaging of Leukocytes

**DOI:** 10.3389/fimmu.2020.02137

**Published:** 2020-09-10

**Authors:** Connie H. Y. Wong, Craig N. Jenne, Elzbieta Kolaczkowska

**Affiliations:** ^1^Centre for Inflammatory Diseases, Department of Medicine, School of Clinical Sciences at Monash Health, Monash University, Clayton, VIC, Australia; ^2^Department of Microbiology, Immunology and Infectious Diseases, University of Calgary, Calgary, AB, Canada; ^3^Department of Experimental Hematology, Institute of Zoology and Biomedical Research, Jagiellonian University, Krakow, Poland

**Keywords:** intravital microscopy (IVM), intravital imaging, leukocytes, inflammation, eye, heart, liver, lymph nodes

The capacity to image motile leukocytes in three dimensions in tissues and organs, *in situ*, over time (the 4th dimension), in a living organism, presents a unique and powerful tool that allows for real-time investigation of their function and behavior. The approach of “seeing” cells in the live animal is called intravital imaging or *in vivo* microscopy (IVM). Recent advancements in microscope technologies, new strains of reporter mice, novel surgical approaches, and broad access to fluorochrome conjugated monoclonal antibodies, have led us to now visualize, at sub-cellular level, deep into living tissues. To reach this stage, many obstacles had to be overcome. For example, imaging of the lung required the opening of the chest cavity without the lung collapsing. Similarly, imaging of organs with strong autonomic innervation (heart, intestines) needs to overcome the dynamic tissue movements. As these technical challenges have been met and overcome, we are now only beginning to reveal the true life of the leukocyte. The goal of this Research Topic was to bring together some of the latest methodological developments in the field of IVM as well as some novel findings both in terms of unstudied diseases, and leukocyte function and cellular fate.

Immune cells play a pivotal role in both maintaining and restoring homeostasis in our bodies. Resident leukocytes are either strategically located in tissues prone to infection (e.g., skin, body cavities), or actively patrol vasculature of some organs (e.g., lung, liver). Once infection or injury occurs, these resident immune cells alert other immune cells, facilitating robust cellular recruitment when needed. In these functions, leukocytes are actively supported by platelets, blood components that in recent years have become highly appreciated as critical helpers and regulators of the immune response. Montague et al. review imaging approaches that have allowed for the study of platelets at the single cell level. These studies have provided great insight into the roles of platelets in both hemostasis and in infection/inflammation, reveling rapid and dramatic shape changes, alterations in adherence and interactions with other blood cells, plasma proteins, and vascular structures. In many respects, the ability to image platelet activation, in real-time, has fundamentally changed our understanding of these multifaceted blood components.

During the inflammatory response to virtually any disorder, the first leukocytes to be recruited to the afflicted tissue are neutrophils. These cells are traditionally regarded as the simple, dispensable foot-soldiers of innate immunity and are largely underappreciated. In this Research Topic, several papers focus on neutrophils, analyzing their behavior and function during sterile (Davis et al.), viral (Pizzagalli et al.), or bacterial (Cichon et al.) inflammation/infection as well as during cancer (Sody et al.). In the latter context, Sody et al. show that tumor associated neutrophils, or TANs, are associated with poor prognosis of cancer, and migrate in a unique fashion into solid tumors via both CXCR2-dependent and -independent mechanisms. Clearly, neutrophils are not only engaged in the first-line of defense but are also involved in adaptive immunity and antigen presentation (1). Pizzagalli et al. report that, upon challenge of mice with attenuated influenza virus (vaccination), neutrophils are “seen” transporting viral particles from the site of infection to the draining lymph nodes. Although lymph node imaging is typically challenging due to their location deep within draining tissues, the rather superficial and accessible location of the popliteal lymph node allows for efficient and clear imaging of this secondary lymphoid tissue, making it one the most frequently imaged lymph nodes [(2), Lopez et al.]. Further developing the ability to image lymph nodes, Lopez et al. have established a method for visualizing the mandibular draining lymph nodes of the eye to study corneal immune responses. It is within these lymph nodes that antigen presentation occurs as inflammation progresses, and it is the dendritic cells (DCs) that play a critical role in lymphocyte activation during the dry eye disease (DED) adaptive immune response. Two papers included in this Research Topic focus on the morphology and kinetics of these DCs in both the eye and lymph nodes of animals affected by DED (Jamali et al.; Ortiz et al.).

Historically, one of the most challenging tissues to image in the live organism is the heart. This difficulty is due to both continual tissue movement (beating) needed to maintain normal tissue perfusion throughout the animal and due to its location deep within the chest. In fact, it has only been in the last decade that high resolution imaging of the living, beating heart of an intact animal has been possible. In this Research Topic, two groups have provided reviews of the key advancements, techniques and knowledge gained from intravital imaging of the heart and the coronary microcirculation with an emphasis on vascular health and leukocyte trafficking (Allan-Rahill et al.; Kavanagh and Kalia). Key in these imaging advancements is the development of a technique that involves the installation of a glass window and a gentle vacuum-mediated tissue stabilization, allowing for fixation of a single field of view for imaging (Allan-Rahill et al.; Kavanagh and Kalia). Although not yet developed for the beating heart, imaging of other tissues can utilize long-term implantable windows, allowing for visualization of a tissue or organ, in a single animal, over an extended period of time (days-weeks), creating the possibility to follow a given pathological process (e.g., tumor progression) over time (3). Sedin et al. developed a system of highly controlled vacuum chambers to stabilize the kidney during IVM for several hours with minimal disruption of local milieu. One of the important, yet not often addressed, shortcomings of window application in IVM is maintenance of the local temperature of the imaged tissue. Ahl et al. constructed a tissue-stabilizing system via 3D printing that aids in the control of tissue temperature underneath the window allowing for imaging of skin or muscle for several hours. The temperature maintenance is also a key methodological issue in the study by Ortiz et al. who have developed an imaging platform heated by circulating water in the region of the lacrimal gland of the eye.

The spectrum of technical issues faced by the IVM users also includes organ autofluorescence. It is an intrinsic parameter that depends on naturally present, endogenous fluorophores, and in the case of the liver, this phenomenon can provide real-time information on the morphology and functional properties of this organ (4). Davis et al. report on strategies to accommodate this autofluorescence and with a methodological approach, they were able to study leukocyte behavior during non-alcoholic fatty liver disease (NAFLD), a disorder rarely studied with IVM due to the high autofluorescence associated with lipidemic liver. The liver is frequently a target for metabolic-induced damage and perturbations as highlighted during the study of another disease affecting this organ, Wilson's disease, resulting from a point mutation leading to copper accumulation in the liver and brain. Cichon et al. contrasted this condition with that of Menkes disease, a condition in which there is not enough copper available. In either case, during sepsis, the organ is affected by either diminished production of neutrophil extracellular traps (NETs) or decreased neutrophil infiltration, respectively, conditions that are readily tracked and characterized using IVM.

In this Research Topic, we present a selection of papers on both latest developments in research on leukocytes in various tissues and organs, along with novel, or improved methodology, for intravital imaging. Studies and reviews presented in this Research Topic cover multiple tissues/organs and they include the ear (Sody et al.), eye (Jamali et al.; Lopez et al.; Ortiz et al.), heart (Allan-Rahill et al.; Kavanagh and Kalia), kidney (Sedin et al.), liver (Cichon et al.; Davis et al.), lymph nodes (Lopez et al.; Pizzagalli et al.), muscle and skin (Ahl et al.). We have come a long way since the nineteenth century when Augustus Waller and Julius Cohnheim observed the vasculature within the tongue of a live frog using very simple light microscopes (5). Contemporary IVM continues to evolve, embracing new technology and methodology that expands the repertoire of organs, deep-set tissues, and cell types that can be imaged as well as analytical tools that enable a better comprehension of obtained data. By embracing these developments, we are now better equipped than ever to reveal the true life of a leukocyte in health and disease.

## Author Contributions

All: inviting contributors, handling and tracking submissions, acting as associate editors for selected manuscripts, inviting reviewers, co-authoring and revising the manuscript. EK: coordinating the team work, drafting the manuscript.

## Conflict of Interest

The authors declare that the research was conducted in the absence of any commercial or financial relationships that could be construed as a potential conflict of interest.

## References

[1] Vono M Lin A Norrby-Teglund A Koup RA Liang F Lore K. Neutrophils acquire the capacity for antigen presentation to memory CD4(+) T cells *in vitro* and *ex vivo*. Blood. (2017) 129:1991–2001. 10.1182/blood-2016-10-744441 28143882 PMC5383872

[2] Secklehner J Lo Celso C Carlin LM. Intravital microscopy in historic and contemporary immunology. Immunol Cell Biol. (2017) 95:506–13. 10.1038/icb.2017.25 28366932 PMC6095455

[3] Alieva M Ritsma L Giedt RJ Weissleder R van Rheenen J Imaging windows for long-term intravital imaging: general overview and technical insights. Intravital. (2014) 3:e29917 10.4161/intv.29917 28243510 PMC5312719

[4] Croce AC De Simone U Freitas I Boncompagni E Neri D Cillo U. Human liver autofluorescence: an intrinsic tissue parameter discriminating normal and diseased conditions. Lasers Surg Med. (2010) 42:371–8. 10.1002/lsm.20923 20583250

[5] Hwa C Aird WC. The history of the capillary wall: doctors, discoveries, and debates. Am J Physiol Heart Circ Physiol. (2007) 293:H2667–79. 10.1152/ajpheart.00704.2007 17693543

